# Geschlechterverteilung in klinischen Studien in der Uroonkologie – Bestandsaufnahme in Deutschland

**DOI:** 10.1007/s00120-026-02875-8

**Published:** 2026-06-29

**Authors:** Marie Christine Roesch, Tabea Walther, Merle Matzen, Nils Gilbert, Daniar Osmonov, Axel Stuart Merseburger, Lea Sophie Lütje

**Affiliations:** Klinik für Urologie, Universitätsklinikum Schleswig-Holstein, Campus Lübeck, Ratzeburger Allee 160, 23538 Lübeck, Deutschland

**Keywords:** Geschlecht, Uroonkologie, Studieneinschluss, Toxizität, Klinische Studie, Dysbalance, Sex, Uro-oncology, Trial participation, Toxicity, Clinical trials, Imbalance

## Abstract

**Hintergrund:**

Während biomarkerabhängige Zulassungen in der Uroonkologie zunehmen, gibt es bislang keine geschlechtsspezifischen Zulassungen trotz entsprechender Subgruppenresultate.

**Ziel der Arbeit:**

Erhebung des Geschlechterverhältnisses in uroonkologischen Studien mit Fokus auf Einschlussrate und Toxizität in Deutschland.

**Material und Methoden:**

Mittels Umfrage unter der AUO der DKG Erfassung folgender Parameter zu Phase-II/III-Studien des Zeitraums 2015–2023 zu den Entitäten Urothelkarzinom (UC) und Nierenzellkarzinom (RCC): Anzahl männlicher/weiblicher Studienpatienten mit CTCAE-I–II- bzw. III–V-Nebenwirkung, Dosismodifikation und Studienabbruch. Vergleich der geschlechtsspezifischen Einschlussraten mit epidemiologischen Benchmarks. Statistische Analysen mittels Fisher’s Exact-Test und Odds Ratios.

**Ergebnisse:**

An 9 rückmeldenden Studienzentren wurden 106 Patient:innen in Studien eingeschlossen, davon waren 20,8 % weiblich. Im Vergleich zu (inter)nationalen Benchmarks erscheinen die Einschlussraten weiblicher Patienten niedriger (RCC: 21,1 % vs. 34,1 % bzw. 26,6 %; UC: 20,7 % vs. 27,0 % bzw. 27,3 %). Dieses gilt über alle RCC-/UC-Tumorstadien hinweg mit Ausnahme des lokal fortgeschrittenen/metastasierten UC – hier ist die Einschlussrate weiblicher Patienten höher (36,4 % vs. 32,5 % bzw. 25,8 %). Tendenziell brechen Frauen häufiger eine Studie ab (54,5 % vs. 33,3 %; *p* = 0,09; OR 2,4). Unterschiede bei Nebenwirkungen und Dosismodifikation waren statistisch nicht signifikant.

**Schlussfolgerung:**

In Deutschland ist eine Dysbalance in RCC-/UC-Studien in den Einschluss- und Abbruchraten zu erkennen, häufiger zum Nachteil weiblicher Patienten. Eine Stratifizierung hinsichtlich des Geschlechts bei Studieneinschluss oder umfangreichere Pharmakokinetikanalysen könnten geschlechtsangepasste Therapiepläne ermöglichen.

**Graphic abstract:**

**Figure:**
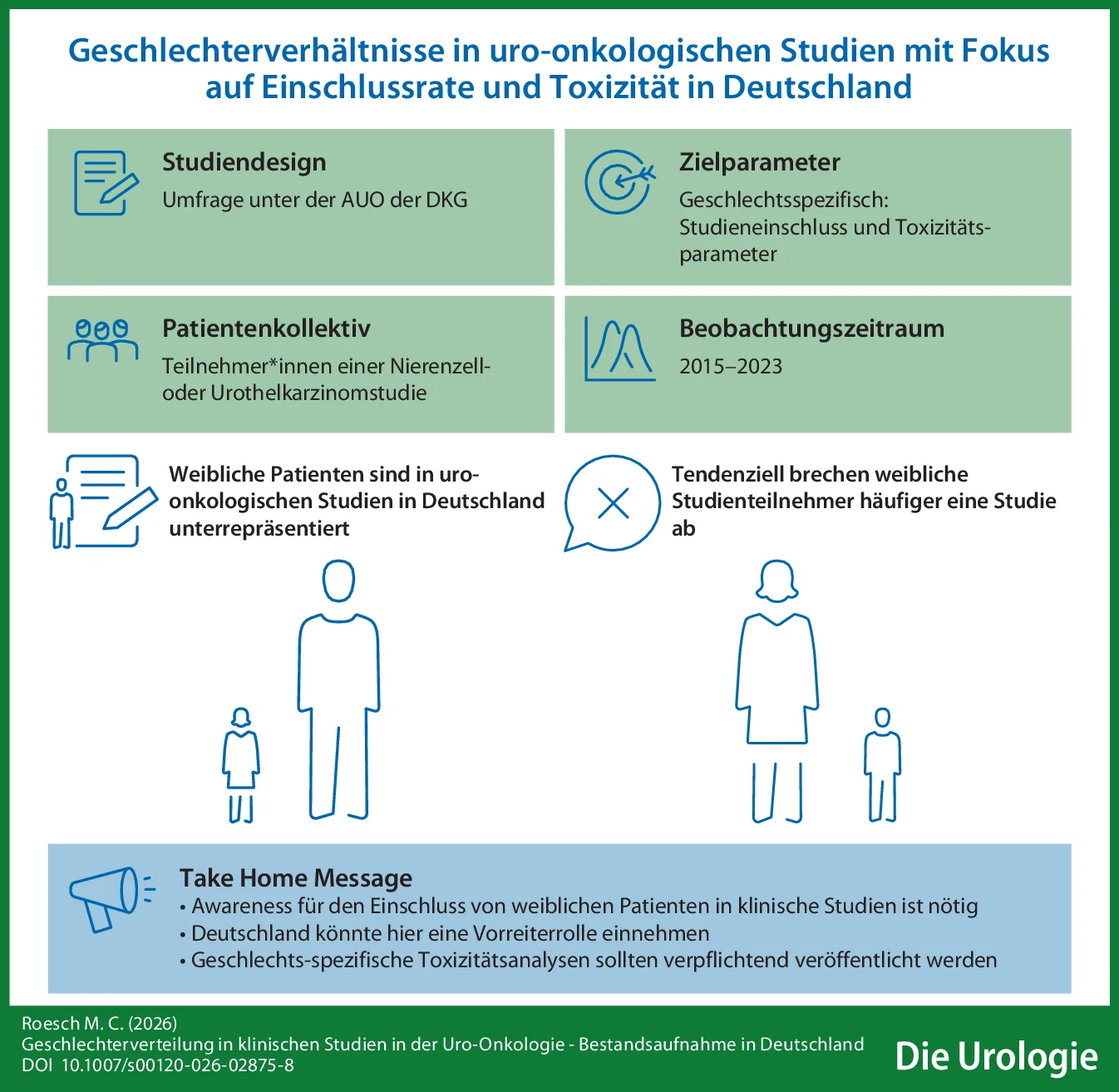

## Abstract (en)

**Background:**

While there are approved biomarker-based drugs in uro-oncology, no sex-specific drug approvals have been granted to date, despite corresponding subgroup results.

**Objective:**

To assess the sex distribution in uro-oncologic clinical trials in Germany, focusing on enrollment rates and treatment-related toxicity.

**Materials and methods:**

A survey was conducted among the Working Group for Urological Oncology (AUO) of the German Cancer Society. The following parameters were collected from phase II/III studies conducted between 2015 and 2023 on urothelial carcinoma (UC) and renal cell carcinoma (RCC): number of male/female study participants with Common Terminology Criteria for Adverse Events (CTCAE) grade I–II or III–V, dose modifications, and study discontinuations. Sex-specific enrollment rates were compared with epidemiological benchmarks. Statistical analyses were performed using Fisher’s exact test and odds ratios.

**Results:**

Across nine responding study centers, 106 patients were enrolled, of whom 20.8% were female. Compared with (inter)national benchmarks, female enrollment rates seemed lower (RCC: 21.1% vs. 34.1% and 26.6%; UC: 20.7% vs. 27.0% and 27.3%). This trend was consistent across all RCC and UC disease stages, except for locally advanced/metastatic UC, where the proportion of females was higher (36.4% vs. 32.5% and 25.8%). Female patients tend to discontinue participation more frequently (54.5% vs. 33.3%; *p* = 0.09; OR = 2.4). Differences in adverse events and dose modifications were not statistically significant.

**Conclusion:**

In Germany, a sex imbalance is evident in RCC and UC clinical trials regarding enrollment and discontinuation rates, often to the disadvantage of female patients. Stratification by sex at trial enrollment or more extensive pharmacokinetic analyses could support sex-adjusted treatment regimens.

Während biomarkerabhängige Zulassungen in der Uroonkologie zunehmen, gibt es bislang keine Geschlechtsspezifischen Zulassungen trotz entsprechender Subgruppenresultate. Zuletzt gab es 2 Schlüsselstudien für das Urothelkarzinom, in der die Subgruppenanalysen geschlechtsspezifische Unterschiede aufzeigen. Diese Arbeit erhebt das Geschlechterverhältnis in uroonkologischen Studien mit Fokus auf Einschlussrate und Toxizität in Deutschland.

## Hintergrund und Fragestellung

In der Uroonkologie gibt es biomarker- oder prognoseabhängige Zulassungen (Beispiele: Pembrolizumab als Erstlinie nur für das lokal fortgeschrittene/metastasierte PD-L1-positive Urothelkarzinom [la/mUC], Nivolumab/Ipilimumab nur für das lokal fortgeschrittene/metastasierte Nierenzellkarzinom mit intermediären oder ungünstigem Risikoprofil [la/mRCC]) sowie körpergewicht- oder metastasenlastabhängige Dosierungsempfehlungen (Beispiel: Enfortumab-Vedotin beim la/mUC je nach Körpergewicht, Denosumab beim Prostatakarzinom je nach Vorliegen von Knochenmetastasen; [1–5]). Für das biologische Geschlecht hingegen gibt es keine Zulassungsbeschränkungen oder Dosierungsunterschiede, obwohl es z. T. ebenso Unterschiede in der Effektivität der Tumortherapeutika gibt. Exemplarisch können hier die EV-301- und NIAGARA-Studie genannt werden [6, 7]. Die EV-301-Studie untersuchte das Antikörper-Wirkstoff-Konjugat Enfortumab-Vedotin (vs. Chemotherapie) an vortherapierten la/mUC-Patienten. Der primäre Endpunkt war das Gesamtüberleben (OS). Die Subgruppenanalyse ergab, dass die HR für männliche Patienten zwar zugunsten Enfortumab-Vedotin ausfiel (HR 0,61; 95 %-Konfidenzintervall [KI] 0,47–0,79), für weibliche Patienten hingegen nicht (HR 1,17; 95 %-KI 0,72–1,89). Die HR für weibliche Patienten lag > 1 und hat ein großes Konfidenzintervall [6]. Das heißt, weibliche Patienten profitierten in dieser Studie nicht von dem untersuchten Medikament. Demgegenüber profitierten in der kürzlich veröffentlichen Auswertung der NIAGARA-Studie weibliche Patienten (HR 0,56; 95 %-KI 0,32–0,94) in Bezug auf das OS (hier sekundärer Endpunkt) von der Hinzunahme von Durvalumab zu neoadjuvanten Chemotherapie beim Blasenkarzinom, während männliche Patienten (in der aktuellen Auswertung noch) nicht profitierten (HR 0,80; 95 %-KI 0,62–1,02; [7]). Dennoch sind beide Therapieregime für beide Geschlechter zugelassen. Weitere Beispiele geschlechtsspezifischer Studienergebnisse sind in einem kürzlich publizierten narrativen Übersichtsartikel unserer Arbeitsgruppe aufgeführt [8].

Es ist bekannt, dass das biologische Geschlecht über die Pharmakokinetik und -dynamik das Therapieansprechen inklusive Toxizität beeinflussen kann. Gründe sind beispielsweise geschlechtsspezifische Unterschiede in (I) der genetischen Variabilität, (II) der Krebsbiologie, (III) dem Körperbau, (IV) der Nierenfunktion, (V) dem Immunsystem, (VI) dem Hormonsystem und (VII) dem Mikrobiom [9, 10]. Hervorzuheben ist, dass auch das Gender sowie das Alter über beispielsweise Compliance, Exposition gegenüber Risikofaktoren, Komorbiditäten und Komedikation Einfluss auf das Therapieansprechen haben [9, 10]. Die dringende Notwendigkeit der geschlechtsangepassten individualisierten Krebstherapie ist eminent. Wie steht es um die Geschlechterverteilung in der klinischen Forschung in Uroonkologie in Deutschland? Das Ziel dieser Bestandsaufnahme ist es, das aktuelle Geschlechterverhältnis in klinischen uroonkologischen Studien mit Fokus auf Einschlussraten und Toxizität an deutschen Studienzentren zu beschreiben. Hierbei werden für diese Arbeit nur UC- und RCC-Studien aus der Uroonkologie bedacht, da die Erkrankungen Peniskarzinom, Hodentumore und Prostatakarzinom nur bei biologisch männlichen Patienten auftreten können. Urethrakarzinome treten insgesamt zu selten auf, als dass eine Inklusion in diese Auswertung sinnvoll wäre.

## Studiendesign

Es erfolgte eine Umfrage per E‑Mail an die Mitglieder bzw. Studienzentren der Arbeitsgemeinschaft Urologische Onkologie (AUO) der Deutschen Krebsgesellschaft e. V. (DKG). Die erste E‑Mail erfolgte am 15.10.2024 mit nachfolgender Erinnerungs-E-Mail am 16.12.2024. Die Datenerhebung wurde am 08.10.2025 beendet (letzte eingegangene berücksichtigte Rückmeldung). Folgendes wurde zu Phase-II/III-Studien, die im Zeitraum 01.01.2015–31.12.2023 an den einzelnen Studienzentren zu den Entitäten UC oder RCC gelaufen sind, abgefragt: Anzahl eingeschlossener männlicher/weiblicher Patienten, Anzahl männliche/weibliche Patienten mit CTCAE I–II oder CTCAE III–V, Anzahl männliche/weibliche Patienten mit Dosismodifikation, Anzahl männliche/weibliche Patienten mit Studienabbruch, Studienname bzw. Studienidentifizierungsnummer. Für die statistische Auswertung der Ergebnisse wurde der exakte Fisher-Test genutzt sowie die Odds Ratio inklusive Konfidenzintervall berechnet (zweiseitige Tests). Als Benchmarks für die Analyse zu den geschlechtsspezifischen Einschlussraten dienen Daten vom RKI (Robert-Koch-Institut), von der IARC bzw. GLOBOCAN (International Agency for Research on Cancer/Global Cancer Observatory) bzw. der Arbeitsgruppe um Rosiello et al. [11–14]. Für die epidemiologische Vergleichszahl bzw. Benchmark zum la/mRCC und la/mUC wurde aus den RKI-Daten jeweils das UICC-Stadium (Union internationale contre le cancer) IV herangezogen, für das lokalisierte RCC die UICC-Stadien I-III, für das NMIBC das UICC-Stadium I und für das MIBC die UICC-Stadien II–III.

Für die Studie liegt ein Ethikvotum der Universität zu Lübeck vor (2024-384_1). Es wurden keine individualisierten Patientendaten, sondern nur studienzentrumspezifische Daten zu einzelnen Studien für diese Auswertung erfasst.

## Ergebnisse

Insgesamt haben 9 Studienzentren geantwortet. An 8 Zentren wurden in dem vorgegebenen Zeitraum 01.01.2015–31.12.2023 insgesamt 30 Phase-II/III-Medikamentenstudien durchgeführt (s. Tab. 1). Ein Studienzentrum hat in dem relevanten Zeitraum keine Phase-II/III-Studie durchgeführt (Zentrum # 4). Eine Phase-III-Studie mit Coaching als Intervention wurde vorab von allen Analysen exkludiert. Von den 30 Studien wurden vier Studien an mehr als einem Zentrum durchgeführt (NIAGARA, MK-866, CONTACT-03 und NIMBUS).

**Tab. 1:** Studiencharakteristika, chronologisch aufgelistet nach rückmeldendem Studienzentrum

Zentrum#	Studie#	Studienkürzel | Studien-ID	Entität | Tumorstadium	Phase
1	1	MK-3475-676-05 | NCT03711032	UC | NMIBC	III
1	2	CA209-901 | NCT03036098	UC (inkl. UTUC) | la/m	III
1	3	PIVOT-10 | NCT03785925	UC (inkl. UTUC) | la/m	II
2	4	JAVELIN Bladder Medley | NCT05327530	UC (inkl. UTUC) | la/m	II
3	5	CA017-078 | NCT03661320	UC | MIBC perioperativ	III
3	6	CA045-009 | NCT04209114	UC | MIBC perioperativ	III
3	7	MK-866 | NCT03924856	UC | MIBC perioperativ	III
3	8	THOR | NCT03390504	UC (inkl. UTUC) | la/m	III
3	9	CaboPOINT | NCT03945773	RCC | la/m	II
3	10	MK-3475-361 | NCT02853305	UC (inkl. UTUC) | la/m	III
3	11	NIMBUS | Eudract Number: 2010-019181-91	UC | NMIBC	III
3	12	MK-3475-426 | NCT02853331	RCC | la/m	III
3	13	MK-3475-564 | NCT03142334	RCC | lokalisiert (adjuvant)	III
4	–	–	–	–
5	14	NIMBUS | Eudract Number: 2010-019181-91	UC | NMIBC	III
5	15	TIM | NCT02106572	UC | NMIBC	III
6	16	CONTACT-03 | NCT04338269	RCC | la/m	III
6	17	RACE IT | ^a^	UC | MIBC perioperativ	II
6	18	NIAGARA | NCT03732677	UC | MIBC perioperativ	III
7	19	NIAGARA | NCT03732677	UC | MIBC perioperativ	III
7	20	VOLGA | NCT04960709	UC | MIBC perioperativ	III
7	21	NeoNIRenCa | EudraCT 2021-005949-33	RCC | lokalisiert (perioperativ)	II
8	22	MK-866 | NCT03924856	UC | MIBC perioperativ	III
8	23	MK-B15 | NCT04700124	UC | MIBC perioperativ	III
8	24	MK-905 | NCT03924895	UC | MIBC perioperativ	III
9	25	SAKK 06/14 | NCT02371447	UC | NMIBC	II
9	26	BLC2001 | NCT02365597	UC (inkl. UTUC) | la/m	II
9	27	TITAN-TCC | NCT03219775	UC (inkl. UTUC) | la/m	II
9	28	CONTACT-03 | NCT04338269	RCC | la/m	III
9	29	IMvigor211 | NCT02302807	UC (inkl. UTUC) | la/m	III
9	30	RANGE | NCT02426125	UC | la/m	III

^a^Keine Eudract/NCT

*la* lokal fortgeschritten („locally advanced“), *m* metastasiert, *MIBC* muskelinvasives Blasenkarzinom, *NMIBC* nicht-muskelinvasives Blasenkarzinom, *RCC* Nierenzellkarzinom („renal cell cancer“), *UC* Urothelkarzinom, *UTUC* Urothelkarzinom des oberen Harntraktes („upper tract urothelial cancer“)

In den 30 gemeldeten Studien wurden insgesamt 106 Patienten eingeschlossen, davon 22 weibliche Patienten (d. h. die Einschlussrate für weibliche Patienten lag bei 20,8 %; Tab. 2). Die Einschlussrate für weibliche Patienten bei separater Betrachtung der Entitäten UC und RCC ist ähnlich (21,1 bzw. 20,7 %). Betrachtet man jedoch die einzelnen Tumorstadien für das UC, so sind hier unterschiedliche Einschlussraten für weibliche Patienten zu verzeichnen: 13,0 % für NMIBC-Studien, 16,6 % für MIBC-Studien und 36,4 % für la/mUC-Studien (Tab. 2). Beim RCC hingegen sind die Einschlussraten für weibliche Patienten über die Tumorstadien hinweg stabil bei 20,0 % bzw. 21,4 %.

**Tab. 2:** Einschlussrate je nach Entität, Tumorstadium und Geschlecht

Einschlussrate je Entität und Tumorstadium	Gesamt (*n*)	Männliche Patienten (*n* [%])	Weibliche Patienten (*n* [%])	Männliche/weibliche Patienten [%]
	Bestandsaufnahme			Benchmarks
RCC und UC gesamt	106	84 (79,2)	22 (20,8)	–
RCC gesamt	19	15 (78,9)	4 (21,1)	RKI [11]: 65,9/34,1 GLOBOCAN [12]: 63,4/36,6
RCC lokalisiert (perioperativ oder adjuvant)	5	4 (80,0)	1 (20,0)	RKI [11]: 65,9/34,1
RCC la/m	14	11 (78,6)	3 (21,4)	RKI [11]: 65,9/34,1 Rosiello et al. [13]: 65,9/34,1
UC gesamt	87	69 (79,3)	18 (20,7)	RKI [11]: 73,0/27,0 GLOBOCAN [12]: 72,7/27,3
UC NMIBC	23	20 (87,0)	3 (13,0)	RKI [11]: 79,6/20,4
UC MIBC (perioperativ)	42	35 (83,3)	7 (16,6)	RKI [11]: 69,9/30,1
UC (inkl. UTUC) la/m	22	14 (63,6)	8 (36,4)	RKI [11]: 67,5/32,5 Rosiello et al. [14]: 74,2/25,8

*la* lokal fortgeschritten („locally advanced“), *m* metastasiert, *MIBC* muskelinvasives Blasenkarzinom, *NMIBC* nicht-muskelinvasives Blasenkarzinom, *RCC* Nierenzellkarzinom („renal cell cancer“), *RKI* Robert-Koch-Institut, *UC* Urothelkarzinom, *UTUC* Urothelkarzinom des oberen Harntraktes („upper tract urothelial cancer“)

**Abb. 1:**
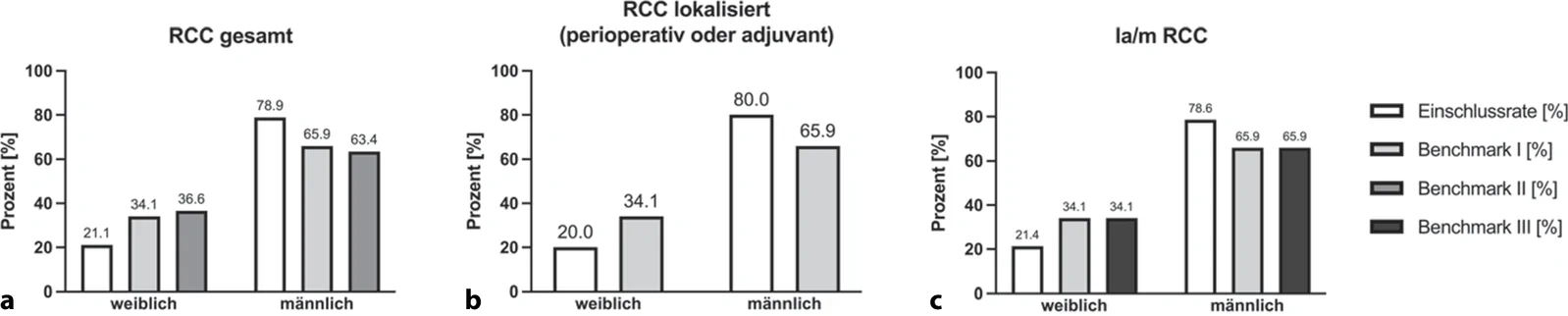
Einschlussrate für RCC-Studien (Nierenzellkarzinom) je nach Tumorstadium und Geschlecht im Vergleich zu Benchmarks (Benchmark I in **a**–**c** Robert-Koch-Institut Krebs in Deutschland [11]; Benchmark II in **a** GLOBOCAN [12], Benchmark III in **c**: Rosiello et al. [13])

Vergleicht man die Studieneinschlussraten mit den nationalen und internationalen epidemiologischen Benchmarks so fällt auf, dass die Einschlussraten weiblicher Patienten in klinischen Studien stadienübergreifend für das RCC und UC unter den Benchmarks zu liegen scheinen (RCC: 21,1 vs. 34,1/26,6 %; UC: 20,7 vs. 27,0/27,3 %; Abb. 1 und 2). Dieses gilt auch für das lokalisierte RCC (20,0 vs. 34,1 %), das la/mRCC (21,4 vs. 34,1/34,1 %), das NMIBC (13,0 vs. 20,4 %) und das MIBC (16,6 vs. 30,1 %). Beim la/mUC scheint die Studieneinschlussrate für weibliche Patienten über den herangezogenen epidemiologischen Benchmarks zu liegen (36,4 vs. 32,5/25,8 %).

**Abb. 2:**
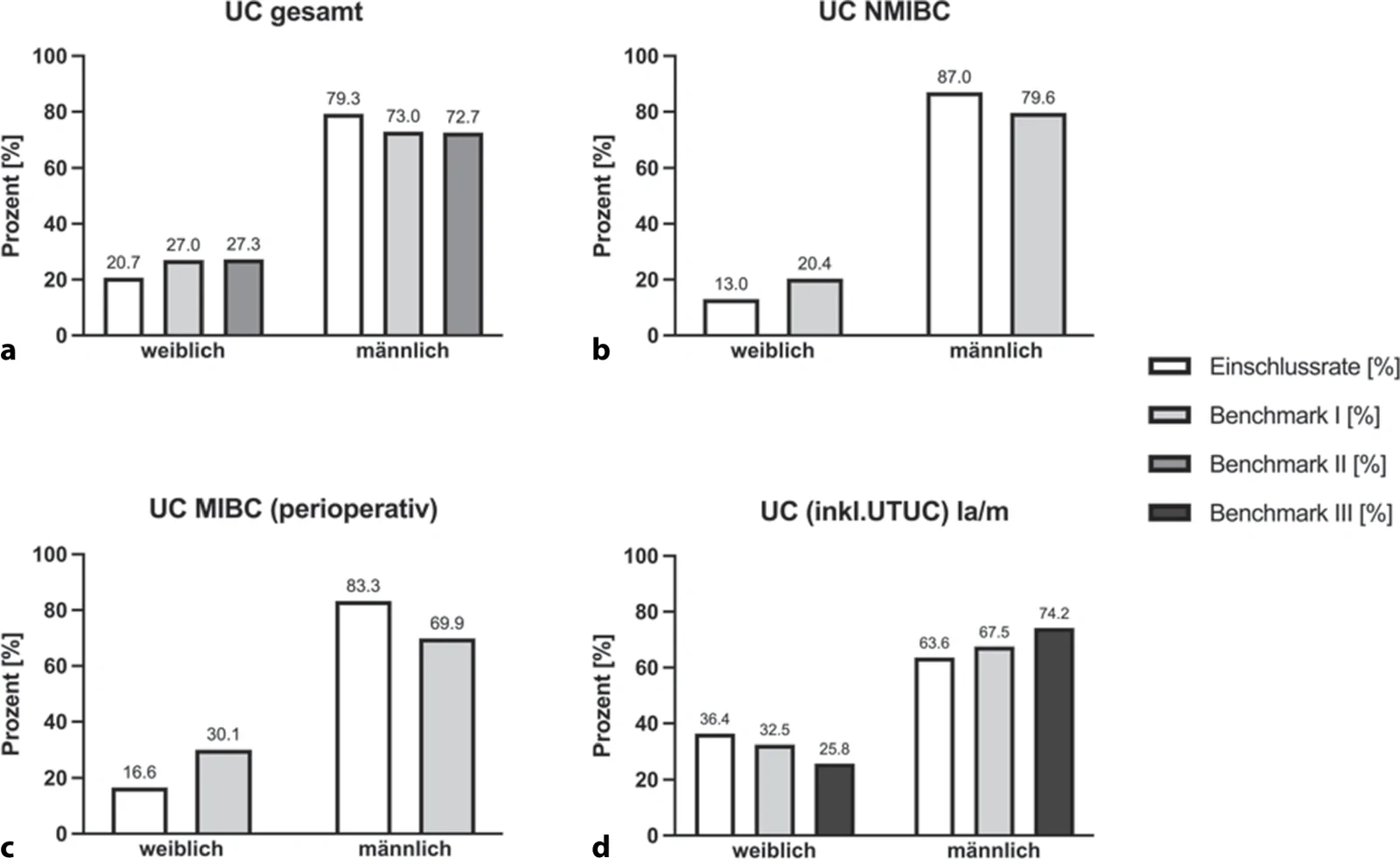
Einschlussrate für UC-Studien (Urothelkarzinom) je nach Tumorstadium und Geschlecht im Vergleich zu Benchmarks (Benchmark I in **a**–**d** Robert-Koch-Institut Krebs in Deutschland [11]; Benchmark II in **a** GLOBOCAN [12], Benchmark III in **d** Rosiello et al. [14])

**Abb. 3:**
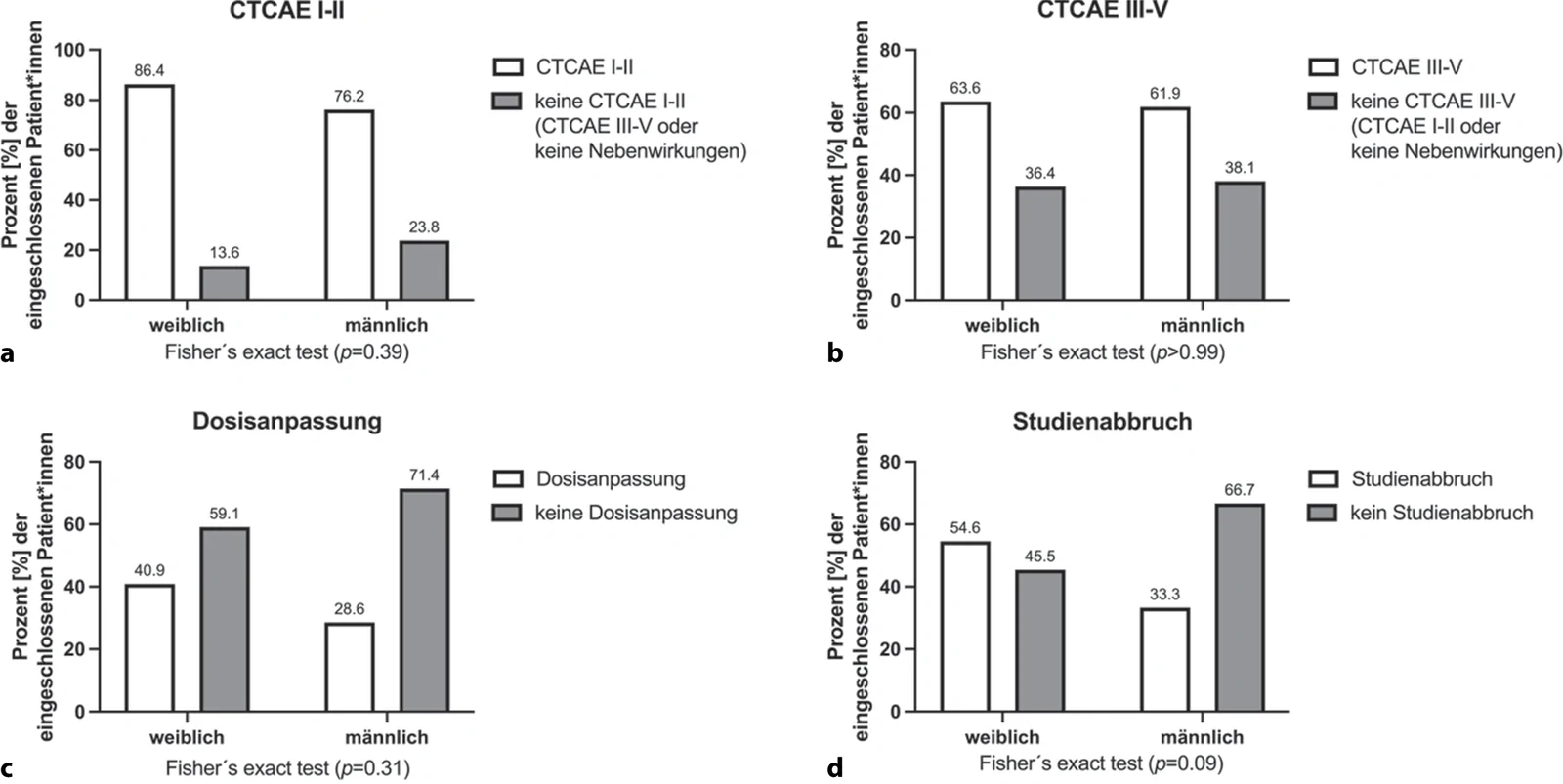
Toxizitätsparameter je nach Geschlecht

In Bezug auf die Toxizitätsauswertungen zeigte sich, dass von den insgesamt 84 eingeschlossenen männlichen Patienten 76,2 % eine unerwünschte Nebenwirkung CTCAE I–II hatten, hingegen 86,4 % der eingeschlossenen 22 weiblichen Patienten (Tab. 3 und Abb. 3, *p* > 0,5). Während 61,9 % der männlichen eingeschlossenen Patienten eine Nebenwirkung CTCAE III–V erfuhren, traf dieses auf 63,6 % der eingeschlossenen weiblichen Patienten zu (*p* > 0,5). Die Dosis musste bei 40,9 % der weiblichen und 28,6 % der männlichen Patienten modifiziert werden (*p* > 0,5). Ein Studienabbruch wurde bei 54,5 % aller weiblichen und 33,3 % aller männlichen Patienten vollzogen (*p* = 0,0854; OR 2,4 mit 95 %-KI 0,9239–5,907).

**Tab. 3:** Toxizitätsparameter je nach Geschlecht

Toxizitätsparameter	Gesamt	Männliche Patienten (*n*)	% von 84 eingeschlossenen männlichen Patienten	Weibliche Patienten (*n*)	% von 22 eingeschlossenen weiblichen Patienten
CTCAE I–II	83	64	76,2	19	86,4
CTCAE III–V	66	52	61,9	14	63,6
Dosismodifikation	33	24	28,6	9	40,9
Studienabbruch	40	28	33,3	12	54,5

## Diskussion

Zusammenfassend zeigt unsere Pilotstudie aus Deutschland auf, dass weibliche Patienten in der Mehrzahl der Fälle im Vergleich zu epidemiologischen Kennzahlenzahlen scheinbar zu selten in klinische Phase-II/III-Studien zum UC und RCC eingeschlossen werden. Einzig beim la/mUC scheint die Studieneinschlussrate weiblicher Patienten über der epidemiologischen Vergleichszahl (d. h. im Umkehrschluss wurden in diesem Fall zu wenig männliche Patienten in die Studien eingeschlossen) zu liegen.

Diese Ergebnisse spiegeln Resultate der Arbeitsgruppe um Kalathoor wider [15]. Kalathoor et al. haben entitätsübergreifend Phase-II/III-Studien zu Tumortherapeutika hinsichtlich der geschlechtsspezifischen Studieneinschlussrate untersucht und haben ihre Zahlen mit US-amerikanischen epidemiologischen Kennzahlen verglichen. Hierbei haben sie genau wie wir nur geschlechts-unabhängige Entitäten berücksichtigt. Unter 149 klinischen Studien mit > 60.000 Teilnehmern waren 40,7 % weiblich (vs. 59,3 % männlich, *p* < 0,01). In 66,9 % der Studien waren jedoch Frauen im Vergleich zu den entitätsspezifischen epidemiologischen Kennzahlen unterrepräsentiert [15]. Eine vergleichbare Analyse führten Dymanus et al. durch [16]. Auch diese Arbeitsgruppe untersuchte 149 onkologische Zulassungsstudien mit ca. 60.000 Patienten und verglich die Einschlussraten mit den krebsspezifischen Inzidenzraten in den USA. In 42 % der Studien waren Frauen unterrepräsentiert. Betrachtet man dezidiert die uroonkologischen Studien in der Analyse so fällt jedoch auf, dass Frauen zwar in Nierenkarzinomstudien (in diesem Fall RCC + UTUC des Nierenbeckens) unterrepräsentiert waren (−7,4 % im Vergleich zur Inzidenz), in den Blasenkarzinomstudien hingegen überrepräsentiert (+10,8 % im Vergleich zur Inzidenz; [16]). Auch Mendis et al. verglichen die geschlechtsspezifischen Einschlussraten onkologischer Studien mit den Inzidenzraten aus den USA und kamen zu gleichen Ergebnissen: entitätsübergreifend war ein Männerüberschuss, bei Karzinomen der Niere ebenso ein Männerüberschuss und beim Blasenkarzinom hingegen erneut ein Frauenüberschuss in den Studien zu verzeichnen [17]. Dieses Phänomen ist auch in unseren Ergebnissen zu la/mUC-Studien zu erkennen. Eine australische Forschergruppe um Merone et al. betrachtete die geschlechtsspezifischen Einschlussraten je nach Fachdisziplin und stellte fest, dass Frauen in den Disziplinen Nephrologie und Kardiologie in klinischen Studien unterrepräsentiert und in den Disziplinen Psychiatrie und Orthopädie überrepräsentiert sind [18]. Es gibt diverse epidemiologische Faktoren, die zu einer höheren Einladungsquote und letztlich dann Teilnahmequote an einer Studie führen können (z. B. höherer Schulabschluss, alleine lebend, in der Stadt wohnend; [19, 20]). Dieses spannende Forschungsfeld sollte für zukünftige Rekrutierungsstrategien mit dem Ziel balancierter Einschlussraten noch weiter exploriert werden.

Unsere geschlechtsspezifische Auswertung zur Studienabbruchrate zeigt auf, dass weibliche Patienten im Trend doppelt so häufig eine Studie vorzeitig beenden wie männliche Studienteilnehmer (OR 2,4 95 %-KI: 0,9239–5,907). Hypothetisch mag diesem Phänomen eine erhöhte Toxizität zugrunde liegen. Numerisch zeigen sich die anderen erhobenen Toxizitätsparameter dieser Auswertung (Nebenwirkungs- und Dosismodifikationsraten) unter den weiblichen Studienteilnehmern erhöht, jedoch ohne statistische Signifikanz. Wie bereits einleitend eingeführt, gibt es geschlechtsabhängige Unterschiede in der Pharmakokinetik und -dynamik. Eine systematische Übersichtsarbeit von Delahousse et al. zeigt Unterschiede in der Pharmakokinetik von Tumortherapeutika zwischen Männern und Frauen. Für folgende uroonkologischen Therapeutika zeigten sich relevante Unterschiede: Atezolizumab, Axitinib, Cabozantinib, Carboplatin und Sunitinib (bzw. der aktive Metabolit). Für all diese Substanzen ist eine niedrigere Clearance bei weiblichen Patienten zu erkennen [9]. Eine niedrigere Clearance führt zu einer höheren Medikamentenexposition mit potentiell höheren Toxizitätsraten. Eine Anpassung im Applikationsschema zur Reduktion von Nebenwirkungen nach bereits erfolgter Zulassung ist durchaus Gegenstand aktueller klinischer Forschung, wie beispielswiese der CABASTY-Trial (angepasste Cabazitaxel-Gabe) beim Prostatakarzinom oder der NIMBUS-Trial (angepasste Bacillus Calmette-Guérin-Gabe) beim NMIBC [21, 22]. Auffallend ist, dass geschlechtsspezifische Toxizitätsraten insgesamt zu wenig bis gar nicht publiziert werden, obwohl nahezu alle (99,2 %) klinischen Studien die Variable Geschlecht erfassen [23]. Die Arbeit von Kalathoor et al. ergab, dass geschlechtsspezifische Toxizitätsergebnisse nur in 4 % aller klinischen Studien zu Tumortherapeutika berichtet wurden [15]. Kürzlich erstellte systematische Übersichtsartikel unserer Arbeitsgruppe zu klinischen Studien zu Systemtherapien für das RCC und UC bestätigen diese niedrige Rate an berichteten geschlechtsspezifischen Toxizitätsanalysen (noch nicht publiziert, eingereicht).

Es sind Limitationen unserer Auswertung zu beachten. Für den direkten Vergleich unserer Daten mit den epidemiologischen Benchmarks war keine statistische Analyse möglich, sodass hier lediglich eine deskriptive Analyse erfolgte. Das RKI nutzt für die differenzierte Betrachtung der Tumorstadien die UICC-Stadien [11]. So enthält das UICC-Stadium IV für das RCC auch nicht-metastasierte T4-Tumoren bzw. die Stadien UICC I-III keine T4-Tumoren. Das UICC-I-Blasenkarzinom enthält nur T1-Tumoren; Ta- und CIS-Tumoren werden in der UICC-Klassifikation nicht bedacht. Im UICC-Stadium IV für das Blasenkarzinom sind auch nicht-metastasierte T4b-Tumoren enthalten, die wiederum in den Stadien UICC II-III fehlen. Die Daten des RKI beinhalten nur das Urothelkarzinom der Harnblase, d. h. sie inkludieren keine UTUC; gleiches gilt für die Daten aus GLOBOCAN [11, 12]. Die Arbeitsgruppe um Rosiello hingegen hat in ihren Analysen ausschließlich mRCC und mUC der Harnblase analysiert (d. h. in beiden Entitäten keine lokal fortgeschrittenen Karzinome und beim UC ebenso keine UTUC; [13, 14]). Die hier erwähnten klinischen Studien zum la/mUC hingegen enthalten in der Regel auch UTUC. Diese Diskrepanz in den einzelnen Definitionen muss bei der Interpretation der Daten berücksichtigt werden. Unsere Abfrage zum Studienabbruch erfasst nicht den Grund des Studienabbruchs. In den meisten Fällen wird dieses sicherlich die Toxizität sein, doch kann eine Studie auch toxizitätsunabhängig z. B. aufgrund eines expliziten Patientenwunsches beendet werden. Die Datenerhebung basiert auf einer retrospektiven Umfrage unter Studienzentren – hierdurch sind Verzerrungen im Sinne eines Selektions- oder Recall-Bias möglich. Es haben nur neun Studienzentren an dieser Umfrage teilgenommen und trotz des langen Befragungszeitraumes wurden nur Daten zu knapp über 100 Patienten übermittelt, beides schränkt die Aussagekraft dieser Studie ein. Die Gesamtanzahl der Empfänger unserer Umfrage war für die Berechnung der Rücklaufquote leider nicht eruierbar. Die Heterogenität der eingeschlossenen Studien hinsichtlich Entität, Tumorstadium und Therapie erlaubt nur eine eingeschränkte Vergleichbarkeit. Insgesamt betonen die oben genannten Limitationen den explorativen Charakter dieser Studie.

## Schlussfolgerung

Diese erste Bestandsaufnahme scheint eine Geschlechterdysbalance in klinischen Studien in der Uroonkologie in Deutschland aufzuzeigen. Diese Dysbalance ist zu Ungunsten weiblicher Patienten in Bezug auf die Studieneinschlussrate und (in der Tendenz) auf die Studienabbruchrate. In zukünftigen klinischen Studien sollte ein besonderes Augenmerk auf eine repräsentative Studieneinschlussrate in Bezug auf das Geschlecht liegen. Möglicherweise kann dieses nur über eine verpflichtende Stratifizierung erfolgen. Studienteams sollten sich dieser aktuellen Geschlechterdysbalance in den Einschlussraten bewusst sein und ggf. weibliche Patienten gezielter für klinische Prüfungen ansprechen. Studienzentralen in Deutschland können hier versuchen, eine Vorreiterrolle einzunehmen. Zusätzlich sollten geschlechtsspezifische Pharmakinetikanalysen in den früheren Phasen einer klinischen Prüfung sowie geschlechtsspezifische Toxizitätsanalysen in Phase-III- und IV-Studien verpflichtend sein. Perspektivisch können solche Maßnahmen zu geschlechtsspezifischen Dosierungsschemata oder Zulassungen führen.

## Fazit für die Praxis

- Eine Awareness für den Einschluss von weiblichen Patienten in klinische Studien ist notwendig.
- Angepasste Rekrutierungsstrategien können zu einem höheren Anteil weiblicher Patienten führen.
- Deutschland könnte hier eine Vorreiterrolle einnehmen.
- Statistische Manöver wie eine Stratifizierung zum Zeitpunkt des Studieneinschlusses können die Geschlechterdysbalance potentiell ausgleichen.
- Geschlechtsspezifische Toxizitätsanalysen können wertvolle Erkenntnisse liefern und sollten verpflichtend veröffentlicht werden.

## Data Availability

Die Rohdaten dieser Arbeit sind sensibel und nur auf Anfrage bei der korrespondierenden Autorin verfügbar.
